# A rabbit model of corneal Ectasia generated by treatment with collagenase type II

**DOI:** 10.1186/s12886-018-0760-z

**Published:** 2018-04-13

**Authors:** Jing Qiao, Haili Li, Yun Tang, Wenjing Song, Bei Rong, Songlin Yang, Yuan Wu, Xiaoming Yan

**Affiliations:** Department of Ophthalmology, Peking University First Hospital, Beijing, 100034 China

**Keywords:** Keratoconus, Corneal ectasia, Collagenase type II, Animal model

## Abstract

**Background:**

To investigate use of collagenase type II for generating a rabbit model of corneal ectasia.

**Methods:**

Ten New Zealand white rabbits were used with right eyes treated as the experimental group and left eyes treated as the control group. After epithelial debridement, a collagenase type II solution (200 μL of 5 mg/mL) was applied in the experimental group at room temperature (24 °C) for 30 min, and a 200 μL solution without collagenase was applied in the control group. Slit-lamp microscopy, the mean keratometry (Km), and central cornea thickness (CCT) were examined before and after the procedure. Corneas were obtained on day 14 for biomechanical evaluation.

**Results:**

No obvious inflammatory reaction was observed in all eyes after the procedure. A statistically significant increase in Km (1.54 ± 1.29D vs − 0.82 ± 0.44D at day7 and 0.89 ± 0.89D vs − 2.11 ± 1.02D at day14) and a statistically significant decrease in CCT (− 23.10 ± 12.17 μm vs 6.20 ± 16.51 μm at day7 and − 16.10 ± 10.46 μm vs 11.60 ± 0.88 μm at day14) were observed in the experimental group compared with the control group. The mean stresses and elastic modulus at 5%, 10%, 15%, and 20% deformities in the experimental group decreased and the differences in elastic modulus between the two groups were statistically significant at 10% and 15% deformities.

**Conclusions:**

Collagenase type II treatment results in mimic KC with increased corneal keratometry and corneal thinning and a lower elastic modulus. An animal model for corneal ectasia can be generated by treatment with collagenase type II.

## Background

Keratoconus (KC) is a non-inflammatory, progressive ectatic disorder, which is usually bilateral, characterized by corneal thinning, scarring, and apical protrusion of the cornea and irregular corneal topography [1, 2]. KC is a common clinical disorder throughout the world, with a reported incidence of approximately 1 per 2000, with no sex or race predilection [3]. This disorder usually begins as a teenager, and often progresses until middle age. KC usually results in progressive visual impairment due to myopia and astigmatism. It is the most common disorder for corneal transplantation in developed countries [4]. Thus, KC has become the focus of extensive clinical and basic research in ophthalmology. The exact etiology of KC is still unknown, although it may involve both genetic and environmental factors [5, 6].

Therapeutic treatments for KC include contact lenses, rigid gas permeable lenses, corneal collagen crosslinking, intracorneal ring segment insertion, and corneal transplantation [7]. Understanding processes involved in disease occurrence and progression can assist in detecting the etiology and treatment of KC. As a valuable and indispensable tool for basic research, in vivo animal models could enable researchers to better understand the pathophysiology of KC and to verify hypotheses of pathogenesis, as well as to evaluate potential treatments. However, no suitable animal model of KC is available, which may be due to its complex and unknown etiology. As a result, there is a need for an animal model that can reproduce the pathophysiological features of this disorder.

KC is the most common corneal ectactic disorder, so it is important to establish a corneal ectasia model to mimic KC. Corneal ectasia can be iatrogenic, which can be a complication of corneal laser refractive procedures [8, 9]. Corneal refractive surgeries remove corneal tissue, disrupt cornea biomechanics, and decrease the collagen tensile strength [10]. Laser in situ keratomileusis (LASIK) on animal corneas, especially rabbits, to develop ectasia has been the most common corneal ectasia animal model [11]. However, post-LASIK ectasia and KC may differ in histopathology and ultrastructure [12]; thus, the post-LASIK ectasia model is not an ideal keratoconic animal model.

An imbalance between degradative enzymes such as acid esterase, acid phosphatase, and cathepsins B and G, and their inhibitors such as corneal inhibitors and macroglobulin is believed to be involved in KC [13–15]. Moreover, studies have reported a reduction in collagen content in the stroma during this disorder [16, 17]. Laboratory studies have reported that collagenase activities increase in organ-cultured KC corneas [16], leading to the possibility that topical collagenase application could be used to generate a corneal ectasia model. Hong et al. reported a significant increase in corneal curvature in human donor corneas after topical collagenase application [18]. In a pilot study, we also observed increased corneal curvature in postmortem collagenase-treated rabbit corneas. However, the supply of collagenase-treated donor corneas is limited. As a result, in the following study, we investigated the use of collagenase type II for generating a rabbit model of corneal ectasia.

## Methods

### Animals and preparation of collagenase type II

Ten female New Zealand white rabbits weighing 3.0–3.5 kg were used in this study. The animals were obtained from Beijing FYY Laboratory Animal Co., Ltd. (Beijing, China) (SCXK 2014–0012). The experimental protocol was approved by the Ethical Committee of Peking University First Hospital. Rabbits were housed in a controlled environment with a 12 h-light/12 h-dark cycle. Food and water were available ad libitum. Continuous clinical care (24 h per day/7 days per week) was provided throughout the study to ensure prompt intervention when needed. Animals were anesthetized intravenously with 0.6 mL/kg of 5% sodium pentobarbital, and 0.4% oxybuprocaine hydrochloride eye drops were used for topical anesthesia during the surgery, as well as pre- and postoperative eye examinations. All of the animals in this study were treated in accordance with the NIH statement for the use of Animals in Research. Collagenase type II (Worthington, Lakewood, NJ, USA) was obtained in powder form and dissolved in balanced salt solution with 15% dextran to a final concentration of 5 mg/mL.

### Surgery

Twenty eyes of 10 rabbits were divided into two groups. The right eyes were treated as the experimental group and the left eyes were treated as the control group. Rabbits were anesthetized intravenously with 0.6 mL/kg of 5% sodium pentobarbital. Topical anesthesia using 0.4% oxybuprocaine hydrochloride eye drops was applied to the eyes. After epithelial debridement, corneal trephines were placed on the corneas. In the experimental group, 200 μL of 5 mg/mL collagenase type II solution was transferred into the corneal trephines, and corneas were immersed in collagenase type II solution at room temperature (24 °C) for 30 min. The solution was then removed by cotton swabs and the corneas were rinsed with 0.9% sodium chloride solution. The control eyes were subjected to the same protocol, but lacking collagenase type II in the applied solution.

### Ophthalmological examinations

Before surgery, the rabbit eyes underwent slit-lamp examinations for the evidence of conjunctival injection, corneal infiltration and cornea stromal inflammation, which were repeated every day during the 14-day study.

### Corneal keratometry

Corneal keratometry was performed on the day before the surgery and 7 and 14 days after the surgery, using a handheld keratometer (Suowei; Tianjin Suowei, Tianjin, China). Eight measurements were taken at each time point and the mean keratometry (Km) and the changes of Km (ΔKm) were recorded in diopters (D).

### Corneal pachymetry

Central cornea thickness (CCT) was recorded on the day before the surgery and 7 and 14 days after the surgery, using a handheld pachymeter (PachPen; Acctome Ultrasound, Malvern, PA, USA) under topical anesthesia. Six measurements were taken at each time point and the mean CCT and the changes of CCT (ΔCCT) were recorded.

### Biomechanical measurements

Rabbits were euthanized with an intravenous overdose of sodium pentobarbital on day 14. The entire cornea with the adjacent sclera (2.0 mm wide) was obtained from each eye. A 4 mm-wide central corneal strip, including 2.0 mm sclera on both ends, was cut by a double-bladed scalpel along the vertical direction (12:00–6:00 o’clock). Corneal strips were fixed in the clamps of a microcomputer-controlled testing machine (Instron 5848 Micro Tester; Instron, Norwood, MA, USA) with a force of 5 N. The corneal strips were stretched at a speed of 3.0 mm/min. Load and deformation were recorded. Elastic modulus is defined as the ratio of tensile stress (amount of force causing deformation per unit trans-sectional area of corneal strips) to the tensile strain (percentage change of the length caused by the stress).

### Histology

The remaining cornea samples were fixed in 4% paraformaldehyde for 3 days and embedded in paraffin. Then they were vertically sliced into sections 8 μm thick, used for hematoxylin-eosin staining.

### Statistical analyses

All results are expressed as the mean ± standard error (SE). Comparisons of changes of Km and CCT between two groups were performed using two-way ANOVA. A two-tailed paired *t*-test was used in the statistical analyses to examine the elastic modulus between two groups. Analyses were performed using SPSS Statistics, version 17.0 (SPSS, Chicago, IL, USA). A value of *P* < 0.05 was considered to be statistically significant.

## Results

### Ophthalmological examinations

After the surgery, daily slit-lamp examinations showed no conjunctival injection, corneal infiltration and cornea stromal inflammation throughout the follow-ups. Fluorescein stain examinations showed complete corneal epithelial healing at approximately 5 days after the surgery.

### Corneal keratometry

**Fig. 1:**
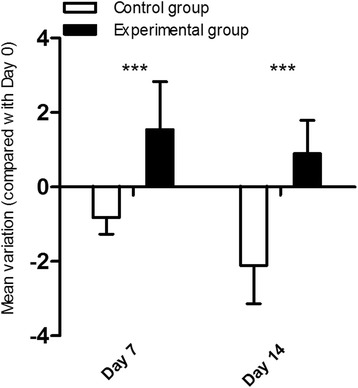
The changes of Km of two groups during the follow-up. There were significant differences between two groups at day 7 and 14

Before the surgery, there was no significant difference in the Km between the two groups (*P* > 0.05). After the surgery, there was an increase in Km in the experimental group and a decrease in Km in the control group. Km in the experimental group increased significantly by 1.54 ± 1.29D and 0.89 ± 0.89D at day 7 and 14 (*P* < 0.05). The changes of Km were significant at both time points (day 7 and 14) compared with the control group (*P* < 0.05) (Fig. 1).

### Corneal pachymetry

**Fig. 2:**
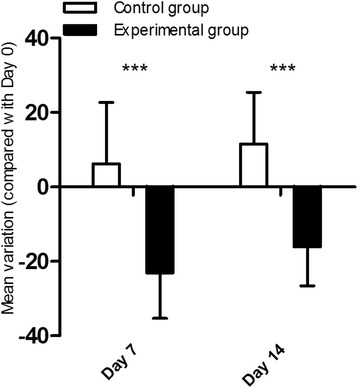
The changes of CCT of two groups during the follow-up. There were significant differences between two groups at day 7 and 14

Before the operation there was no significant difference in the CCT between the two groups (*P* > 0.05). CCT in the experimental group decreased significantly by − 23.10 ± 12.17 μm and − 16.10 ± 10.46 μm at day 7 and 14 (*P* < 0.05). The changes of CCT were significant at both time points (day 7 and 14) compared with the control group (*P* < 0.05) (Fig. 2).

### Biomechanical measurements

**Fig. 3:**
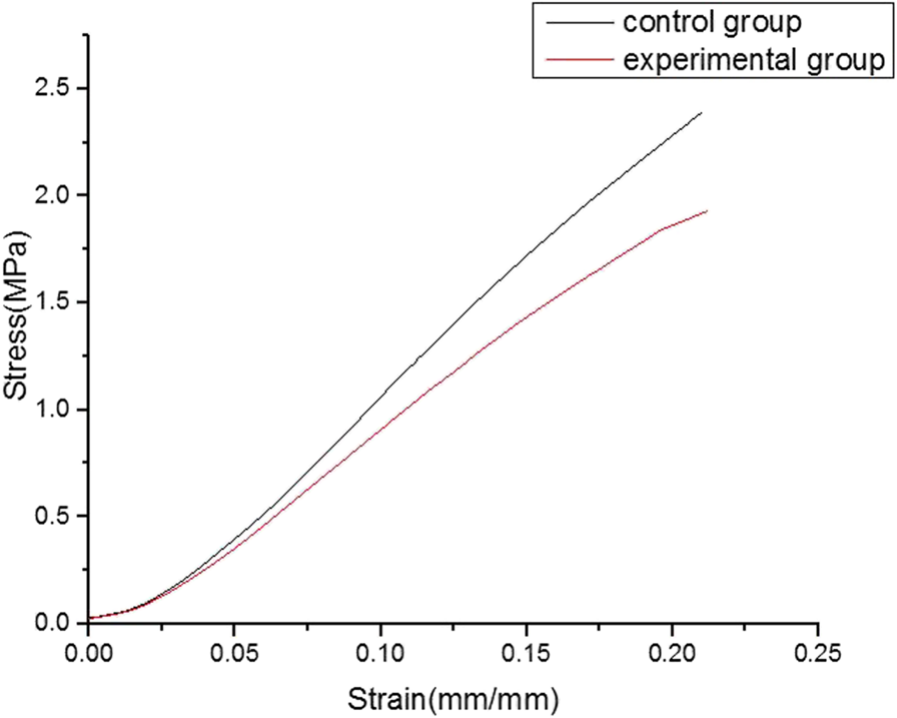
Stress-strain curves in corneal strips. The stress-strain curves of strips treated with collagenase were gentler than those of untreated strips

The stress–strain measurements showed a decrease in the stiffness of the corneas exposed to collagenase. The mean stresses at 5%, 10%, 15%, and 20% deformities were 0.40 ± 0.18 MPa, 1.06 ± 0.32 MPa, 1.69 ± 0.23 MPa, and 2.23 ± 0.20 MPa for the control corneas, and 0.35 ± 0.17 MPa, 0.91 ± 0.25 MPa, 1.43 ± 0.20 MPa, and 1.77 ± 0.20 MPa for the treated corneas, respectively, with a decrease of 11.64%, 14.74%, 15.37%, and 17.95%, respectively. The differences between the two groups were not significant (all, *P* > 0.05) (Fig. 3). After collagenase treatment, the elastic modulus of the corneas was significantly lower. The elastic modulus of the treated corneas at 5%, 10%, 15%, and 20% deformities were lower than that of control corneas, and the differences between the two groups were statistically significant at 10% and 15% deformities (*P* < 0.05) (Table 1).

**Table 1:** Comparison of the elastic modulus in collagenase-treated and control corneas

Strain	Elastic modulus (Mpa)		*P*
	Experimental group	Control group	
5%	10.23 ± 4.50	11.43 ± 5.00	0.614
10%	11.18 ± 1.56	13.99 ± 1.52	0.048^*^
15%	9.46 ± 4.68	12.22 ± 4.66	0.015^*^
20%	7.02 ± 1.79	9.09 ± 0.86	0.125

^*^P < 0.05

### Histology

**Fig. 4:**
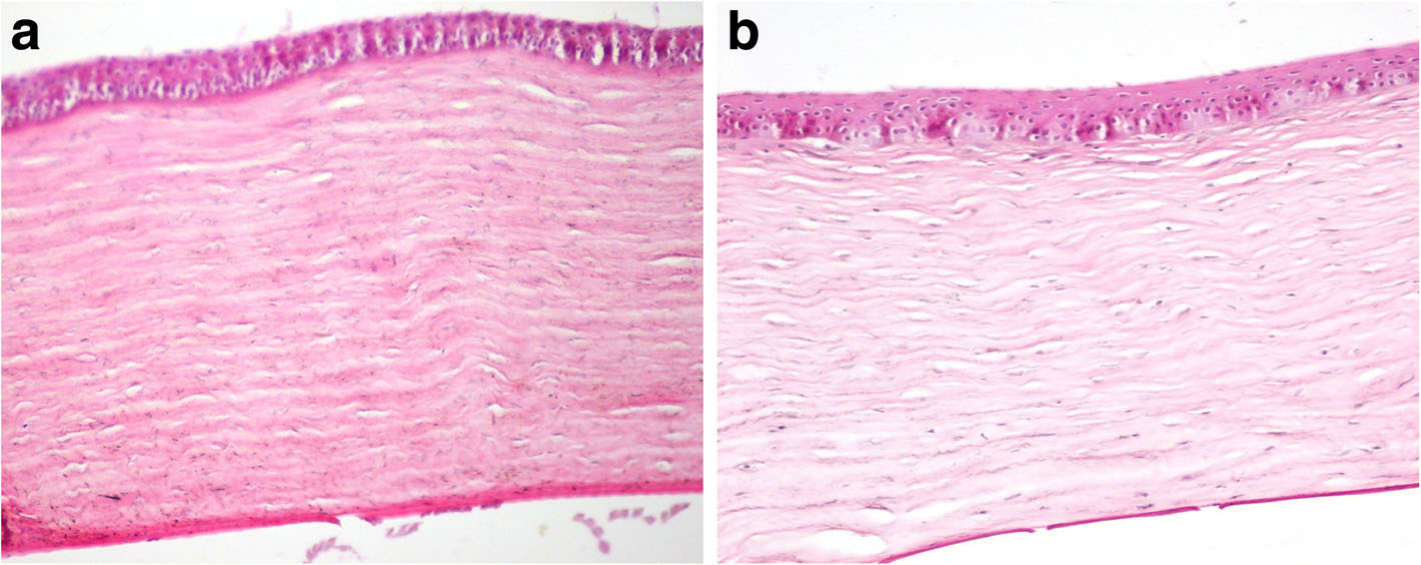
Hematoxylin-eosin stained corneal sections. Compared to the control group (**a**), collagen fibers in the experimental group (**b**) appeared more loosely arranged and interlamellar clefts appeared

Compared to the control group (Fig. 4a), collagen fibers in the experimental group appeared more loosely arranged and interlamellar clefts were observed (Fig. 4b).

## Discussion

KC is a corneal ectatic disease of unknown etiology characterized by irregular corneal topography and progressive thinning and steeping of the cornea. However, progresses in basic research and clinical treatments have been limited by the lack of a suitable animal model for this disorder. It has been postulated that weakening of the cornea induced by abnormal structure and/or composition play an important role in the development of KC [3]. Decreased collagen lamellae, abnormal organization of the collagen fiber network, and loss of collagen fibrils in the stroma are observed in keratoconic corneas. These processes may be related to the abnormal regulation of collagenase, protease, and inhibitors of matrix metalloproteinase (MMP)-1 and MMP-3 in the degradative pathway of collagen [19–21]. It has been reported that abnormal corneal structure and composition also contribute to the biomechanical failure that characterizes the pathological process of KC. It is therefore possible that a keratoconic model can be generated by degrading collagens. Collagenase is a member of the MMP family of enzymes that degrade collagen [22]. Collagenase type II preferentially degrades collagen I, which is the main collagen component of cornea; thus, it is possible that exposure to collagenase can generate a corneal ectasia model.

Previous studies have used an animal model of corneal ectasia involving removal of excess corneal stroma, to produce a postsurgical corneal ectasia [23]. Ectasia after LASIK surgery is thought to be caused by failure of structural integrity due to excess tissue removal or to the presence of subclinical KC [24]. As previously mentioned, abnormal structure, abnormal composition, and biomechanical weakness due to the abnormal degradative pathway of collagen may play a major role in KC. The pathogenesis of ectasia after LASIK surgery may therefore differ from KC. Furthermore, previous studies have reported histopathological and ultrastructural differences between ectasia after LASIK surgery and KC [12]. Although ectasia after LASIK surgery and KC both have fewer and thinner lamellae, the lamellae only exist in the residual stromal bed in ectasia after LASIK surgery, but are more widely present in KC. In addition, corneal thinning during ectasia after LASIK surgery is mainly due to tissue removal, which does not occur in KC. Ectasia after LASIK surgery therefore cannot be regarded as a suitable model for KC.

In the present study, collagenase treatment induced a significant increase in Km (1.54D and 0.89D) and a significant decrease in CCT (23.1 μm and 16.1 μm) at day 7 and 14, respectively, which is consistent with the clinical characteristics of irregular corneal topography and progressive corneal thinning. Other studies have shown the similar findings ex vivo. Hong CW et al. reported that collagenase treatment resulted in a significant increase in corneal curvature in human donor corneas by 6.6 ± 1.1D [18]. Wang X et al. demonstrated that rabbit corneas exposed to collagenase ex vivo showed a significant decrease in CCT [25]. Interestingly we found time also had a significant influence in Km in two-way ANOVA analyses. Some studies also focus on the relationship between the corneal keratometry and time. Angunawela RI et al. reported that normal rabbit corneas flattened and had a reduction in the mean keratometry of − 1.9 ± 1.0D over 56 days [26]. Kompa S et al. also demonstrated a continuous decrease of refractive power in rabbits by about 2D in 26 days [27]. Doughty MJ et al. explained that this decrease of refractive power was a natural aging phenomenon caused by the growth of rabbit’s globe [28]. Our study also revealed similar decrease of Km in the control group. When considering the natural flattening of the cornea and the reduction in keratometry in both eyes, the total keratometry of the experimental eyes increased by more than 2D. Moreover, the increase in keratometry and decrease in CCT after collagenase exposure lasted for 2 weeks, suggesting that the rabbit model of corneal ectasia generated by collagenase treatment support good short-time feasibility for the future evaluation of novel therapies of KC.

As previously mentioned, biomechanical failure plays a major role in pathogenesis of KC. Numerous studies comparing biomechanical properties between keratoconic and normal corneas reported lower rigidity, increased stress, increased strain, and increased energy absorption in keratoconic corneas [14, 29]. As an indicator of material stiffness, the elastic modulus is usually used to evaluate the biomechanical properties of keratoconic corneas. Relative to normal corneas, some studies have reported reductions in the elastic modulus in keratoconic corneas [29–31]. The finite element model of KC has been considered a tool for theoretical analyses of the geometry and optical responses to biomechanical alterations and corneal thinning. Gefen et al. reported that corneal thinning combined with a lower elastic modulus, but not corneal thinning alone, could produce forward displacement and peak dioptric values consistent with clinical cases of KC in a finite element model of KC [32]. They concluded that corneal thinning and reduction in the elastic modulus are both involved in the pathogenesis of KC. Histopathology revealed more loosely arranged collagen fibers and interlamellar clefts after collagenase treatment.

In the present study, tensile testing of corneal strips showed that the elastic modulus of corneas exposed to collagenase were significantly lower compared with that of the controls, resulting in both corneal thinning and reduced tensile strength. Furthermore, no obvious inflammatory reactions were observed in the eyes throughout the follow-ups, indicating that treatment with collagenase does not result in inflammation.

Our study did not examine the long-term effects of this novel corneal ectasia model, which is the main limitation. First of all, this 2-week observation may be too short for a comprehensive understanding of this enzymatic degradation model, although in the present study it had shown a significant increase in Km and significant decreases in CCT and elastic modulus in 2 weeks. Besides, long-time observation is necessary for further identification the changes of Km, CCT and tensile strength, which could confirm the sustainability of this novel model. Additional studies with long-time observation are warranted to better investigate the feasibility and efficacy of this rabbit model of corneal ectasia in vivo. The development of novel acute animal model has a great potential for assessing the experimental therapies for KC. Fourteen days may be sufficient for an acute cornea ectasia model to preliminarily evaluate novel treatments for KC after careful review of other studies that develop novel in vivo corneal models [33, 34]. Our findings obtained in 2 weeks showed that collagenase treatment may provide a successful model of corneal ectasia.

## Conclusions

Our study described a rabbit model of corneal ectasia involving collagenase treatment, which simulated KC in corneal topography, corneal thinning, and a lower elastic modulus. The model can be used to further investigate the pathogenesis of KC and to evaluate novel treatments for this disorder.

## Abbreviations

CCT: Central cornea thickness
KC: Keratoconus
Km: Mean keratometry
LASIK: Laser in situ keratomileusis

## References

[1] Katsoulos C, Karageorgiadis L, Vasileiou N, Mousafeiropoulos T, Asimellis G (2009). Customized hydrogel contact lenses for keratoconus incorporating correction for vertical coma aberration. Ophthalmic Physiol Opt.

[2] Krachmer JH, Feder RS, Belin MW (1984). Keratoconus and related noninflammatory corneal thinning disorders. Surv Ophthalmol.

[3] Ambekar R, Toussaint KC, Wagoner Johnson A (2011). The effect of keratoconus on the structural, mechanical, and optical properties of the cornea. J Mech Behav Biomed Mater.

[4] Rabinowitz YS (1998). Keratoconus. Surv Ophthalmol.

[5] Abu-Amero KK, Al-Muammar AM, Kondkar AA: Genetics of keratoconus: where do we stand? J Ophthalmol 2014, 2014:641708. 10.1155/2014/641708 PMC4164130 25254113

[6] Nielsen K, Hjortdal J, Pihlmann M, Corydon TJ (2013). Update on the keratoconus genetics. Acta Ophthalmol.

[7] Espandar L, Meyer J (2010). Keratoconus: overview and update on treatment. Middle East Afr J Ophthalmol.

[8] Seiler T, Koufala K, Richter G (1998). Iatrogenic keratectasia after laser in situ keratomileusis. J Refract Surg.

[9] Tervo TM (2001). Iatrogenic keratectasia after laser in situ keratomileusis. J Cataract Refract Surg.

[10] Binder PS (2007). Analysis of ectasia after laser in situ keratomileusis: risk factors. J Cataract Refract Surg.

[11] Liu YC, Konstantopoulos A, Riau AK, Bhayani R, Lwin NC, Teo EP, Yam GH, Mehta JS (2015). Repeatability and reproducibility of corneal biometric measurements using the Visante Omni and a rabbit experimental model of post-surgical corneal Ectasia. Trans Vis Sci Technol.

[12] Dawson DG, Grossniklaus HE, McCarey BE, Edelhauser HF (2008). Biomechanical and wound healing characteristics of corneas after excimer laser keratorefractive surgery: is there a difference between advanced surface ablation and sub-Bowman's keratomileusis?. J Refract Surg.

[13] Collier SA (2001). Is the corneal degradation in keratoconus caused by matrix-metalloproteinases?. Clin Exp Ophthalmol.

[14] Kenney MC, Brown DJ, Rajeev B (2000). Everett Kinsey lecture. The elusive causes of keratoconus: a working hypothesis. CLAO J.

[15] Collier SA, Madigan MC, Penfold PL (2000). Expression of membrane-type 1 matrix metalloproteinase (MT1-MMP) and MMP-2 in normal and keratoconus corneas. Curr Eye Res.

[16] Kao WW, Vergnes JP, Ebert J, Sundar-Raj CV, Brown SI (1982). Increased collagenase and gelatinase activities in keratoconus. Biochem Biophys Res Commun.

[17] Mackiewicz Z, Maatta M, Stenman M, Konttinen L, Tervo T, Konttinen YT (2006). Collagenolytic proteinases in keratoconus. Cornea.

[18] Hong CW, Sinha-Roy A, Schoenfield L, McMahon JT, Dupps WJ (2012). Collagenase-mediated tissue modeling of corneal ectasia and collagen cross-linking treatments. Invest Ophthalmol Vis Sci.

[19] Sawaguchi S, Yue BY, Chang I, Sugar J, Robin J (1991). Proteoglycan molecules in keratoconus corneas. Invest Ophthalmol Vis Sci.

[20] Meek KM, Tuft SJ, Huang Y, Gill PS, Hayes S, Newton RH, Bron AJ (2005). Changes in collagen orientation and distribution in keratoconus corneas. Invest Ophthalmol Vis Sci.

[21] Sawaguchi S, Yue BY, Sugar J, Gilboy JE (1989). Lysosomal enzyme abnormalities in keratoconus. Arch Ophthalmol.

[22] Overall CM, Lopez-Otin C (2002). Strategies for MMP inhibition in cancer: innovations for the post-trial era. Nat Rev Cancer.

[23] Riau AK, Liu YC, Lwin NC, Ang HP, Tan NY, Yam GH, Tan DT, Mehta JS (2014). Comparative study of nJ- and muJ-energy level femtosecond lasers: evaluation of flap adhesion strength, stromal bed quality, and tissue responses. Invest Ophthalmol Vis Sci.

[24] Roberts CJ, Dupps WJ (2014). Biomechanics of corneal ectasia and biomechanical treatments. J Cataract Refract Surg.

[25] Wang X, Huang Y, Jastaneiah S, Majumdar S, Kang JU, Yiu SC, Stark W, Elisseeff JH (2015). Protective effects of soluble collagen during ultraviolet-a crosslinking on enzyme-mediated corneal Ectatic models. PLoS One.

[26] Angunawela RI, Riau AK, Chaurasia SS, Tan DT, Mehta JS (2012). Refractive lenticule re-implantation after myopic ReLEx: a feasibility study of stromal restoration after refractive surgery in a rabbit model. Invest Ophthalmol Vis Sci.

[27] Kompa S, Ehlert E, Reim M, Schrage NF (2000). Microbiopsy in healthy rabbit corneas. A long-term study. Acta Ophthalmol Scand.

[28] Doughty MJ (1994). The cornea and corneal endothelium in the aged rabbit. Optom Vis Sci.

[29] Andreassen TT, Simonsen AH, Oxlund H (1980). Biomechanical properties of keratoconus and normal corneas. Exp Eye Res.

[30] Edmund C (1989). Corneal topography and elasticity in normal and keratoconic eyes. A methodological study concerning the pathogenesis of keratoconus. Acta Ophthalmol Suppl.

[31] Nash IS, Greene PR, Foster CS (1982). Comparison of mechanical properties of keratoconus and normal corneas. Exp Eye Res.

[32] Gefen A, Shalom R, Elad D, Mandel Y (2009). Biomechanical analysis of the keratoconic cornea. J Mech Behav Biomed Mater.

[33] Mello GR, Pizzolatti ML, Wasilewski D, Santhiago MR, Budel V, Moreira H (2011). The effect of subconjunctival bevacizumab on corneal neovascularization, inflammation and re-epithelization in a rabbit model. Clinics.

[34] Gronkiewicz KM, Giuliano EA, Kuroki K, Bunyak F, Sharma A, Teixeira LB, Hamm CW, Mohan RR (2016). Development of a novel in vivo corneal fibrosis model in the dog. Exp Eye Res.

